# An Immunohistochemical Study on the Role of CD83+ Dendritic Cells (DCs) in Malignant and Benign Lesions of the Human Cervix

**DOI:** 10.7759/cureus.71327

**Published:** 2024-10-12

**Authors:** Kalpana Ramachandran

**Affiliations:** 1 Anatomy, Sri Ramachandra Institute of Higher Education and Research, Chennai, IND

**Keywords:** cd83, cervix, dendritic cell, hysterectomy, squamous cell carcinoma

## Abstract

Background

Dendritic cells (DCs) are a group of cells that mainly function as antigen-presenting cells in the human body. Proper knowledge and understanding of such cells in the human cervix would be beneficial for understanding the role of CD83+ cells in benign and malignant lesions of the cervix.

Materials and methods

This retrospective study was performed on cervical specimens. After processing, the CD83+ cells were counted for every 20 high-power fields. The average count per high power field (HPF) was then calculated. The CD83+ cell distributions in cervicitis, cervical dysplasia, and cervical carcinoma were then analyzed.

Results

A total of 30 cervical specimens were studied. Of these, 16 were cervicitis and seven were squamous cell carcinoma. Vaginal bleeding was the most common presentation in 21 patients. The mean age was 44.7 years. The mean CD83+ DCs in benign lesions was 1.75 and in malignant tissues was 12.26 per HPF (P<0.001). The area under the curve suggested a 100% sensitivity and specificity of CD83 in distinguishing benign and malignant lesions. The receiver operating characteristic (ROC) curve indicated that the probability of malignancy is higher if the number of CD83+ DCS is more than 179.50/20 HPF.

Conclusions

Dendritic cells play a major role in the tumoricidal activities of the host cervical tissues. Malignant cervical tissue possesses a higher concentration of CD83+ DCs than benign ones, with 100% sensitivity and specificity. This research work on CD83+ DCs in the cervix would pave the way for further research on the immune functions of the human body.

## Introduction

Dendritic cells (DCs) are the white blood cells that guard the human body by acting as surveillance cells of our immune system. These heterogenous accessory cells act as a specific form of antigen-presenting cells of human host immunity. Whenever a foreign antigen infects the human cells, these DCs facilitate the presentation of such antigens to the host immune system, thereby immediately destroying them [1]. DCs are mostly concentrated around the mucosal surface lining epithelial cells. The main function of these cells would be to initiate the host's innate immunity and also to augment the immune responses against the antigens that either invade or the host cells that undergo mutation [2].

The DCs originate from the progenitor cells of bone marrow and slowly migrate toward the lymphoid tissues and lymphocytes. Upon reaching the lymphocytes, they stimulate the T-helper cells and initiate action against the foreign antigens [3]. These DCs are further subclassified into myeloid and lymphoid cells, based on their path and mode of development [4,5]. Thus, they act as policemen of the host immune tissue, thereby identifying the mutant cells, capturing them, presenting them to the host immune lymphoid tissue, and destroying them.

CD83 is an immunohistochemical marker that is specific for mature dendritic cells. As a member of the glycoprotein family, these CD83 cells upregulate during the process of maturation of DCs. The mature DCs show an elaborated expression of CD83, CD80, and CD86, while immature cells express CD1a [6]. Therefore, the mature and immature dendritic cell populations can be well studied by looking for and identifying the CD83 and CD1a, respectively [7].

Cervical cancers are one of the leading cancers that affect women. Worldwide, about 600,000 cervical cancer cases and nearly 350,000 cancer-specific deaths occur every year [8]. Ever since Dai et al. studied the ultrastructure of the Langerhans cells in the human cervix, the research on the DCs of the human cervix gained momentum [9]. DCs have been considered and accepted over time as one of the most potent antigen-presenting cells in human tissues. Their uncanny ability to migrate towards the antigenic focus is considered to be the key factor in the initiation and maintenance of immune surveillance and tolerance [10]. The mature CD83 cells have a good migratory ability and exhibit a higher expression of molecules needed for a good antigen presentation. On the other hand, the immature DCs have restricted mobility and form a dense network across tissue interfaces and the external environment [11].

Various studies have demonstrated the increased concentration of dendritic cells in higher grades of cervical dysplasia [12,13]. However, Hughes et al. observed a reduced number of DCs in higher grades of cervical dysplasia [14]. They observed a reduction in the number of epithelial Langerhans cells and altered morphology of DCs in high-grade cervical dysplasia.

Researchers studied the role of DCs in the cervix and the human uterus. However, there is not much published literature evidence to demonstrate the real pattern and dispersal of CD83+ DCs in the human cervix. In this manuscript, we studied the pattern and distribution of CD83+ DCs in benign and malignant lesions of the cervix using immunohistochemical techniques.

Objectives

To study the overall number and density of CD83+ DCs in the cervical tissue using various immunohistochemical (IHC) techniques; to assess and analyze the pattern of distribution of the CD83+ dendritic cells in cervical specimens; to study the significance of the differential pattern of CD83 densities in benign and malignant tissues of the cervix; and to see if we can assess the reliability of CD83 as an immunohistochemical marker in facilitating the diagnosis of benign or malignant pathology in patients with indeterminate cervical biopsy reports.

## Materials and methods

This retrospective study was done on 30 cervical specimens. The institutional ethical committee clearance was obtained from Sri Muthu Kumaran Medical College and Research Institute, Chennai, India, where the author was earlier working (IEC: 05/2015). The period of study was five years, from 2016-2020. Benign and malignant cervical tissues were collected from the pathological specimens of women aged 35 to 50 years who either were subjected to radical hysterectomy for cancer cervix or who underwent cervical biopsy to diagnose cervical pathology. Specimens were processed using the histological techniques that are routinely used. Tiny sections measuring as thin as 3 μm were made and 3μm thick sections of each were obtained on routine and charged slides (coated with poly-L-lysin). Routine slides were stained with H&E, and for charged slides, immunohistochemical staining for CD83+ DCs was done. A technician with 15 years of experience cut the tissues, processed, and immune-stained the slides. This maneuver greatly aided in antigen identification in formalin-fixed and paraffin-embedded tissue. The technique used for immunohistochemical staining was the Mouse/Rabbit Polymer-HRP/DAB detection system, which is a biotin-free detection system. The primary antibody used was monoclonal mouse anti-human CD83. The positive control used was skin with dendritic cells.

DCs were found in abundance in cervical tissue. All CD83+ cells were counted for every 20 high-power fields, using 40X for the objective and 10X for the eyepiece. The average count of CD83+ cells per high-power field was then done. Those nucleated cells with highly discernible processes that protrude from each cell were called the fundamental anatomical arrangements of the DCs. All slides were reviewed by two senior reviewers. High-definition cameras were used to capture images. IBM SPSS Statistics for Windows, Version 20 (Released 2011; IBM Corp., Armonk, New York, United States) software was used to analyze the statistical data. Descriptive variables including median, range, median, and standard deviation were studied. The abnormal dendritic cells in cervical dysplasia and squamous cell carcinoma of cervix specimens were compared using Fischer’s exact test and student-independent t-test.

Inclusion criteria

Patients who underwent total abdominal hysterectomy for various benign clinical conditions like fibroid uterus, endometriosis, and adenomyosis, women who had a cervical biopsy done for making a diagnosis of cervical intra-epithelial neoplasia, non-specific cervicitis, and hyperplasia or dysplasia of the cervix, and patients who undergo radical hysterectomy for endometrial carcinoma or cancer of the cervix were included in our study.

Exclusion criteria

Patients who are immunocompromised, those who have undergone chemotherapy and radiation for other malignancies, and those patients unwilling to participate in this study were excluded from the study.

## Results

About 30 specimens were studied. Table 1 demonstrates the demographic details of all 30 specimens.

**Table 1:** Demographic profile of the specimens studied.

Demographic details of the specimens studied (n=30)	
Pathological condition n (%)	
Chronic nonspecific cervicitis	16 (53.3)
Basal cell hyperplasia	1 (3.3)
Hyperplasia with atypia	1 (3.3)
Carcinoma in situ	2 (6.7)
Chronic inflammation/squamous metaplasia	3 (10)
Squamous cell carcinoma	7 (23.3)
Clinical presentation n (%)	
Bleeding	21 (70)
Lower abdominal pain	4 (13.3)
Mass abdomen	3 (10)
Mass descent per vaginum	2 (6.7)
Age at presentation (in years)	
Minimum	36
Maximum	55
Mean	44.7
Median	48

More than half (n=16) were chronic non-specific cervicitis. Seven of them were malignant. The stratified squamous epithelium that lines the normal cervical tissue contained CD83+ dendritic cells in all specimens.

Table 2 illustrates the comparison of CD83 positivity in benign and malignant cervical tissues. The mean CD83+ DCs per high power field (HPF) in the benign group was 1.75 cells/HPF, and that in the pre-malignant and malignant groups was 17.02 cells/HPF. This data shows that the number of CD83+ DCs increases in cancer tissues compared to benign cervical specimens. The difference between the two groups was statistically significant (P< 0.0001). 

**Table 2:** Comparison of the CD83+ DCs of the benign and malignant lesions of the cervix using the Mann-Whitney U test. CIN: cervical intraepithelial neoplasia; DC: dendritic cells; HPF: high power field

No of CD83+ DCs/HPF (N=30)	Histological type	Mean DCs per HPF	95% CI (Degree of freedom)	p-value
	Nonspecific cervicitis (n=16)	1.75	0.0426-0.5983	< 0.0001
	Dysplasia/CIN/squamous cell carcinoma (n=14)	17.02		

**Figure 1:**
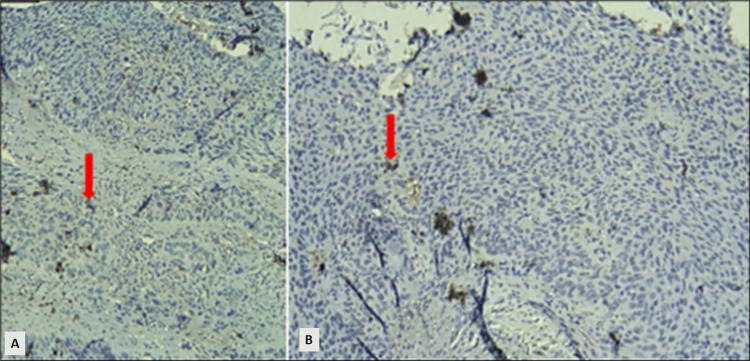
Microphotograph of CD83+ dendritic cells in the specimen of differentiated squamous cell carcinoma, infiltrating. Figures A and B represent the CD83+ dendritic cells (red arrows).

The dendritic cell count was done using an Olympus BX40 microscope (Olympus Corporation, Japan) at a magnification of 40X objective and 10X eyepiece. The number of CD83+ cells was counted per 20 high-power fields (40X objective and 10X eyepiece). Figure 1 shows CD83+ dendritic cells in the infiltrating differentiated squamous cell carcinoma. Sheets and nests of squamoid cells exhibiting moderate to marked pleomorphism and hyperchromasia with areas of keratinization were observed.

**Figure 2:**
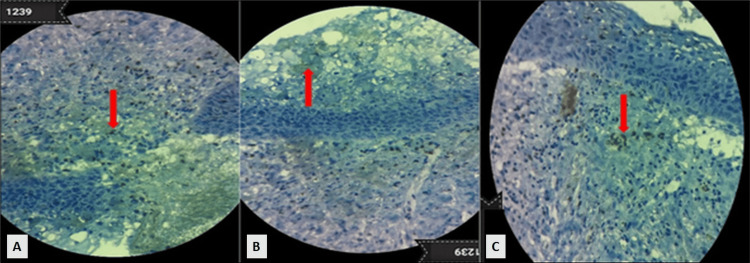
Microphotograph of CD83+ dendritic cells in the specimen of carcinoma in situ cervical specimen. Figures A, B, and C represent the CD83+ dendritic cells (red arrows).

Figure 2 shows an ecto cervix with a moderate degree of dysplasia (cervical intraepithelial neoplasia (CIN) II). CD83+ dendritic cells are seen in the CIN II ectocervical epithelium, which shows basal-like cells, the proliferation of cells with enhanced mitotic activity, and an increased nuclear-cytoplasmic ratio percolating through the middle of the epithelium.

**Figure 3:**
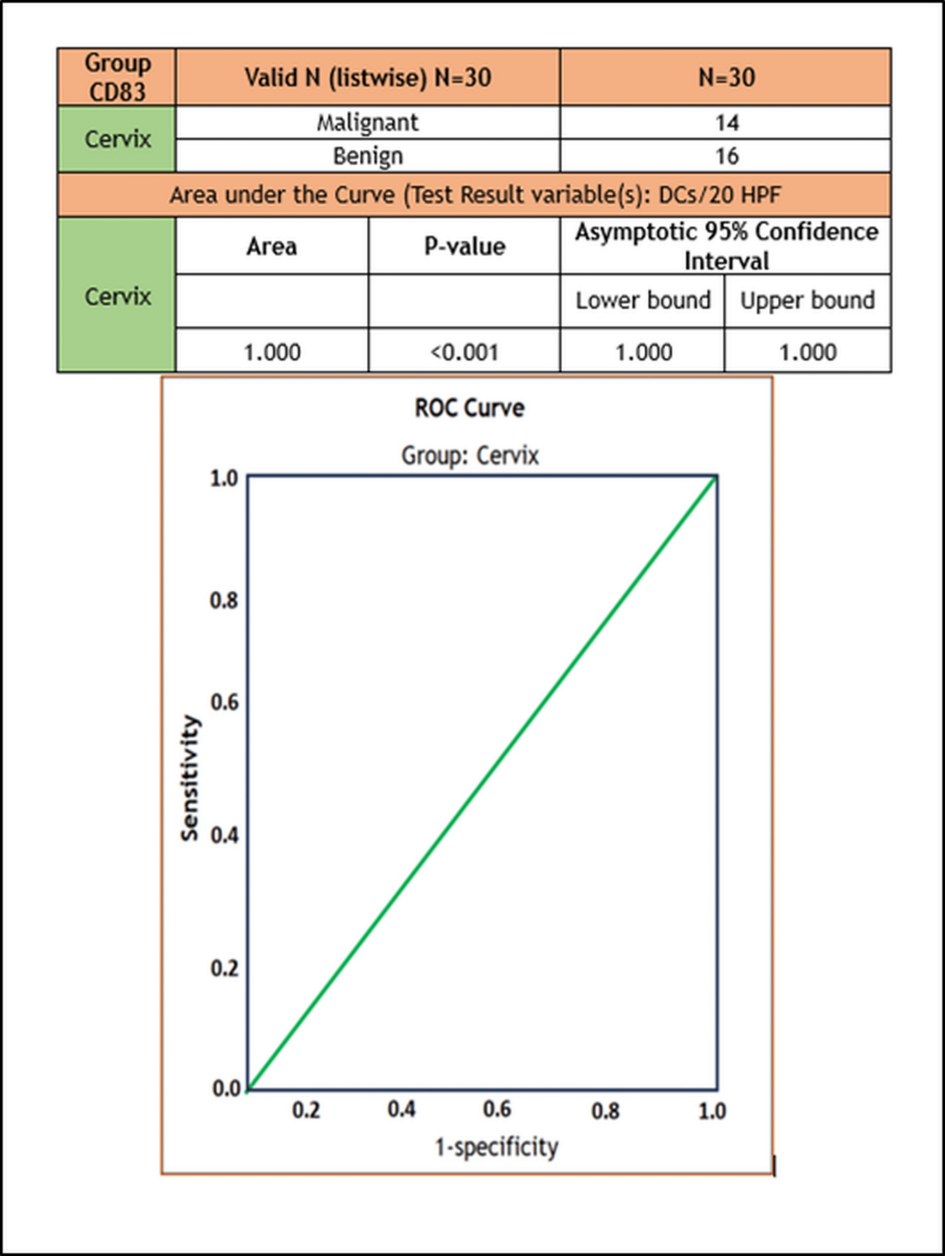
Area under the curve depicting sensitivity and specificity of the CD83+ DC count. DC: dendritic cells

Figure 3 illustrates the area under the curve for the CD83+ 30 cervical specimens. The specificity of CD83 in identifying the benign and malignant cervical specimens is 100%. This means that the CD83 positivity would be able to make a distinction between the benign and malignant specimens with the highest degree of accuracy in all 30 specimens.

Table 3 illustrates the receiver operating characteristic (ROC) curve of the CD83+ DCs in cervical tissues. The ROC curve is used to assess the overall diagnostic performance of a test. It is mainly used to select an optimal cut-off value for determining the presence or absence of a disease. From the above table, the ROC curve suggests that if the number of DCs per 20 HPF is above or equal to 179.50/20 HPF, the probability of the cervical specimen harboring malignancy is high. The sensitivity and specificity are 100%.

**Table 3:** Receiver operating characteristic (ROC) curve of the CD83+ DCs in cervical specimens, showing the cut-off values for true positive and true negative results. DC: dendritic cells; HPF: high power field

CD83	Number of DCs per 20 HPF	Sensitivity	1-Specificity
Cervix	9.00	1.00	1.000
	11.00	1.00	.938
	13.00	1.00	.875
	16.00	1.00	.813
	36.50	1.00	.375
	39.00	1.00	.313
	41.50	1.00	.250
	47.00	1.00	.188
	53.50	1.00	.125
	57.50	1.00	.063
	179.50	1.00	0.000
	190.00	.929	0.000
	200.00	.857	0.000
	200.00	.786	0.000
	220.00	.714	0.000
	180.00	.643	0.000
	340.00	.571	0.000
	387.00	.143	0.000
	387.00	.071	0.000
	457.00	0.000	0.000

The area under the ROC curve is an appropriate way to give a summary of the overall diagnostic accuracy of that particular test. The values range from 0 to 1. A value of 0 indicates a highly inaccurate test, and a value of 1 suggests a perfectly accurate test. A high sensitivity denotes a higher rate of true positivity (when a test is positive, the disease exists indeed). From the above table, the DC count below the cut-off value indicates a higher probability of an underlying benign disease.

The term “1-specificity” means the probability that a true negative will test positive. If 1-specificity is zero, that means that all true negatives are indeed disease-free. From the above table, the cut-off value is 179.50 DCs per 20 HPF. This means that any DC count above this cut-off value will suggest a higher probability of an underlying malignancy. 

## Discussion

Dendritic cells are distinct cells that play an essential role in the commencement of immune reactions. The surface antigen cells execute this function with a high degree of precision. The female reproductive tract harbors a high density of such DCs. According to their embryological origin, the DCs can be classified into myeloid and lymphoid cells. Plasmacytoid group of cells are those lymphoid variety DCs that aid in recognizing the foreign body and initiate the immune response through the elaboration of Type I interferons. The T-helper response is mediated by the myeloid group of DCs [15].

Human papillomavirus (HPV) is a viral infection that is a causative factor in the development of cervical cancer. The viral load may be inadequate to initiate and further progress the lesion. The antigen-presenting cells give protection to the keratinocytes that are infected by the HPV, thereby enhancing the innate and adaptive immunity of the host tissues.

Table 4 gives the cut-off values for making a diagnosis in each of the tissues studied. The CD1a for the uterus and cervix and CD83 for the uterus were studied by the authors earlier. From this table, we observe that the number of DCs is higher in the cervix than in the uterus, irrespective of whether they are benign or malignant.

**Table 4:** Cut-off values, comparison with author’s previous studies. HPF: high power field; PPV and NPV: positive and negative predictive values; AUC: area under the curve

Specimen	Cut-off value for diagnosing malignancy (DCS/20 HPF)	AUC	P-value	Sensitivity	Specificity	PPV	NPV	% false positive	% false negative
CD1A uterus [16]	<6.5	1.000	<0.001	100	100	100	100	0	0
CD1A cervix [17]	<144.5	0.951	<0.001	100	87.5	87.5	100	12.5	0
CD83 uterus [18]	>159.5	1.000	<0.001	100	100	100	100	0	0
CD83 cervix (present study)	>179.50	1.000	<0.001	100	100	100	100	0	0

The area under the curve is constantly 1 for all except CD1a cervix specimens. This means that the sensitivity and specificity are 100%, making a diagnosis of benign or malignant tissues based on the CD count in CD1a uterus, CD83 uterus, and CD83 cervix tissues.

All four staining methods were able to identify and distinguish benign and malignant uterine and cervical specimens with the highest degree of accuracy. The difference between the DC distribution in the two different pathologies has been statistically significant (P<0.001).

Palucka removed the contaminating cells and studied the number of mature DCs after overnight cultivation. The immature DCs matured after an overnight culture, resulting in a 10-time rise in CD83+ DCs [19]. The shorter culture time raised doubts that the monocytes may not have sufficient time to get differentiated enough to express CD83. Based on his double-fluorescence analysis, Zhou further clarified that the monocytes were not activated into CD83 cells [20]. It's possible that the low count of CD83 cells, as discovered by the fluorescence-activated cell sorting (FACS) technique, is a part of the DCs originating from the skin [21].

The CD83 cells are reliable cell surface markers for mature DCs. Wakabayashi studied the effect of CD83 cells on the augmentation of cellular immunity [22]. He also concluded that the lack of CD83 expression diminished the expression of the allogenic T-cell-mediated immune system. 

Furihata studied the plausible role of CD83 in prognostication in patients with gall bladder carcinoma [23]. He found that patients with an elevated CD83 count performed better than those with low counts in combating the antigenic stimuli. In our study, the presence of a higher concentration of CD83+ DCs in malignant tissues predicts a better prognosis. Researchers have also attempted to correlate laryngeal papilloma with CD83+ DCs. However, there is very limited literature available on the quantity and distribution of CD83+ DCs in cervical neoplasia as against the malignant cervical epithelium or endometrium. Our study observed a higher CD83 concentration in patients with uterine cancer and endometrial cellular atypia. Bell observed a higher CD83 concentration in the peri-tumoral area and a higher CD1a concentration within the tumor tissues [7]. However, in our study, we observed abundant CD1a and a lesser concentration of CD83 cells in benign tissues of the endometrium and cervix. 

Therapeutic DNA vaccines against bacterial and viral infections enhance the longevity of humans. Further studies are needed to analyze the use of the antigen-presenting ability of such DCs in vaccine production and immune therapies [24].

Limitations of our study

A larger sample size with multi-center involvement would make this data more authentic. The ROC curves were drawn on a sample size of 30. The values would be more accurate if the sample size was much larger. A comparative study of CD1a and CD83 would throw more light on the pattern and distribution of mature and immature dendritic cells in the various pathological conditions of the cervix. 

## Conclusions

Dendritic cells play a major role in the tumoricidal activities of the host cervical tissues. Malignant cervical tissue possesses a higher concentration of CD83 cells than benign ones. The ROC curves show 100% sensitivity and specificity for the CD83+ DCs. Also, the positive predictive value for CD83+ DCs in malignant cervical tissue is 100%. This research work on the distribution of the CD83+ dendritic cells in cervical tissues would pave the way for further research on the immune functions of the human body.
